# Effect of Live Poultry Market Interventions on Influenza A(H7N9) Virus, Guangdong, China

**DOI:** 10.3201/eid2212.160450

**Published:** 2016-12

**Authors:** Jie Wu, Jing Lu, Nuno R. Faria, Xianqiao Zeng, Yingchao Song, Lirong Zou, Lina Yi, Lijun Liang, Hanzhong Ni, Min Kang, Xin Zhang, Guofeng Huang, Haojie Zhong, Thomas A. Bowden, Jayna Raghwani, Jianfeng He, Xiang He, Jinyan Lin, Marion Koopmans, Oliver G. Pybus, Changwen Ke

**Affiliations:** Guangdong Provincial Center for Disease Control and Prevention, Guangzhou, China (J. Wu, J. Lu, X. Zeng, Y. Song, L. Zou, L. Yi, L. Liang, H. Ni, M. Kang, X. Zhang, G. Huang, H. Zhong, J. He, X. He, J. Lin, C. Ke);; University of Oxford, Oxford, UK (J. Lu, N.R. Faria, T.A. Bowden, J. Raghwani, O.G. Pybus);; Erasmus Medical Center, Rotterdam, the Netherlands (M. Koopmans)

**Keywords:** Influenza virus, A(H7N9), live-poultry market, phylogeography, phylogeny, viruses, China, influenza

## Abstract

Since March 2013, three waves of human infection with avian influenza A(H7N9) virus have been detected in China. To investigate virus transmission within and across epidemic waves, we used surveillance data and whole-genome analysis of viruses sampled in Guangdong during 2013–2015. We observed a geographic shift of human A(H7N9) infections from the second to the third waves. Live poultry market interventions were undertaken in epicenter cities; however, spatial phylogenetic analysis indicated that the third-wave outbreaks in central Guangdong most likely resulted from local virus persistence rather than introduction from elsewhere. Although the number of clinical cases in humans declined by 35% from the second to the third waves, the genetic diversity of third-wave viruses in Guangdong increased. Our results highlight the epidemic risk to a region reporting comparatively few A(H7N9) cases. Moreover, our results suggest that live-poultry market interventions cannot completely halt A(H7N9) virus persistence and dissemination.

## Abstract 2

Temporary closure of these markets appears not to have halted virus transmission or prevented its dissemination.

Since its first notification on March 30, 2013 (*1*), avian influenza A(H7N9) virus caused 3 complete epidemic waves of human infection in China, comprising 670 laboratory-confirmed clinical cases and 274 deaths as of December 28, 2015 (http://www.wpro.who.int/outbreaks_emergencies/H7N9/en/). Despite the accumulating number of human cases, how this virus disseminated and transmitted across the 3 epidemic waves is not yet understood.

Direct or indirect prior exposure to live poultry or poultry-related environments is the major risk for A(H7N9) infection in humans (*2*,*3*). In response to the A(H7N9) outbreak, major efforts were undertaken to temporarily close and sanitize live poultry markets (LPMs) in epicenter cities during epidemics (*4*–*6*). These interventions are thought to temporarily decrease A(H7N9) contamination of LPM environmental samples (*4*,*6*) and to reduce the incidence of clinical infection (*3*,*6*). However, whether these viruses persist locally across epidemic waves despite current interventions has yet to be answered.

Guangdong Province in southern China accounts for ≈10% of China’s domestic poultry industry and is thought to be an important epicenter of influenza A virus circulation (*7*). The province reported no clinical cases during the first wave of A(H7N9) infection but represented the epicenter of the second and third epidemic waves. We integrated epidemiologic, spatial, and genetic data to trace the temporal and spatial origins of influenza A(H7N9) in humans during 2013–2015 in Guangdong.

## Materials and Methods

### Ethics Statement

The institutional ethics committee of the Center for Disease Control and Prevention of Guangdong Province (Guangdong CDC) approved this study. Written consent was signed by patients or their guardian(s) when samples were collected. Patients were informed about the study before providing their written consent, and the data were anonymized for analysis.

### Influenza A(H7N9)Surveillance and Sequencing

Since the first A(H7N9) case in late March 2013, an enhanced provincial surveillance program in all 21 prefecture-level cities in Guangdong has been conducted by a total of 871 clinics and 21 local centers for disease control. All specimens from persons with suspected A(H7N9) infections were tested for subtypes H5, H7, and H9 as previously described (*8*,*9*).

In April 2013, Guangdong CDC launched an environmental surveillance program to monitor avian influenza viruses in LPMs (*9*). Environmental samples were collected from LPMs in Guangdong Province during April 15, 2013–May 30, 2015 (*8*–*10*). Ten to 20 environmental samples per market were collected from selected markets in 21 prefecture-level cities. When A(H7N9) infection was confirmed in a person in a given location and that person had exposure to a specific LPM, at least 20 environmental samples were collected from that market within 24 hours after confirmation of human infection.

Upon reverse transcription PCR testing, H7N9- and H9N2-positive swab materials and sputum samples from patients and LPM environments were blindly passaged for 2–3 generations in 9- to 10-day-old embryonated chicken eggs for virus isolation. All 8 segments of the selected isolates were sequenced by using a next-generation sequencing strategy for influenza A virus with the Ion PGM System and PathAmpFluA Reagents (Life Technologies, Carlsbad, CA, USA). Specific primer sets were used to amplify and fill potential gaps (*8*,*9*).

### Sequence Alignment and Maximum-Likelihood Phylogenetic Analysis

A total of 1,124 nt sequences were generated by this study. These sequences were combined with all publicly available complete gene sequences of influenza A viruses with known sampling dates and locations that belong to subtypes H7N9, H9N2, and other closely related subtypes. Multiple sequence alignment was performed by using ClustalW (*11*), and alignments were minimally edited by using Aliview (*12*). Maximum-likelihood trees were estimated for all 8 gene segments (hemagglutinin [HA], n = 865; neuraminidase [NA], n = 788; nucleoprotein [NP], n = 1,879; basic polymerase proteins 1 and 2 [PB1 and PB2], n = 1,773 and n = 1,826, respectively; polymerase, n = 1,839; matrix, n = 1,841; and nonstructural [NS], n = 838) in RaxML (*13*) by using the generalized time-reversible nucleotide substitution model with gamma -distribution among site rate heterogeneity (*14*). For each gene dataset, we assessed temporal accumulation of genetic divergence from the root-to-tip from maximum-likelihood midpoint-rooted phylogenies using TempEst (formerly Path-O-Gen) (*15*).

### Dated Phylogenetic Analysis

To infer dated phylogenetic trees in a reasonable computational time, we reduced the size of our datasets by removing identical sequences collected in the same sampling locations on the same date. We also removed H9N2 sequences that were phylogenetically unrelated to the H7N9 sequences in our study but kept all H7N9 sequences from clinical cases in Guangdong. Bayesian Markov chain Monte Carlo (MCMC) inferences were undertaken by using BEAST, using a SRD06 nucleotide substitution model (*16*), a relaxed molecular clock model with an uncorrelated lognormal rate distribution (*17*), and a Bayesian skygrid coalescent model (*18*). Four independent MCMC runs of 1 × 10^8^ steps were computed and ≈10%–15% burn-in was discarded, resulting in ≈3.5 × 10^8^ total steps for each gene dataset. Parameters and trees were sampled every 35,000th and 70,000th MCMC step, respectively. Convergence of MCMC chains was inspected by using Tracer version 1.6 (http://tree.bio.ed.ac.uk). A subset of 500 trees was drawn randomly from the combined posterior distribution of trees and used as an empirical distribution for subsequent analysis (*19*).

### Spatial and Temporal Origins of H7N9

**Figure 1:**
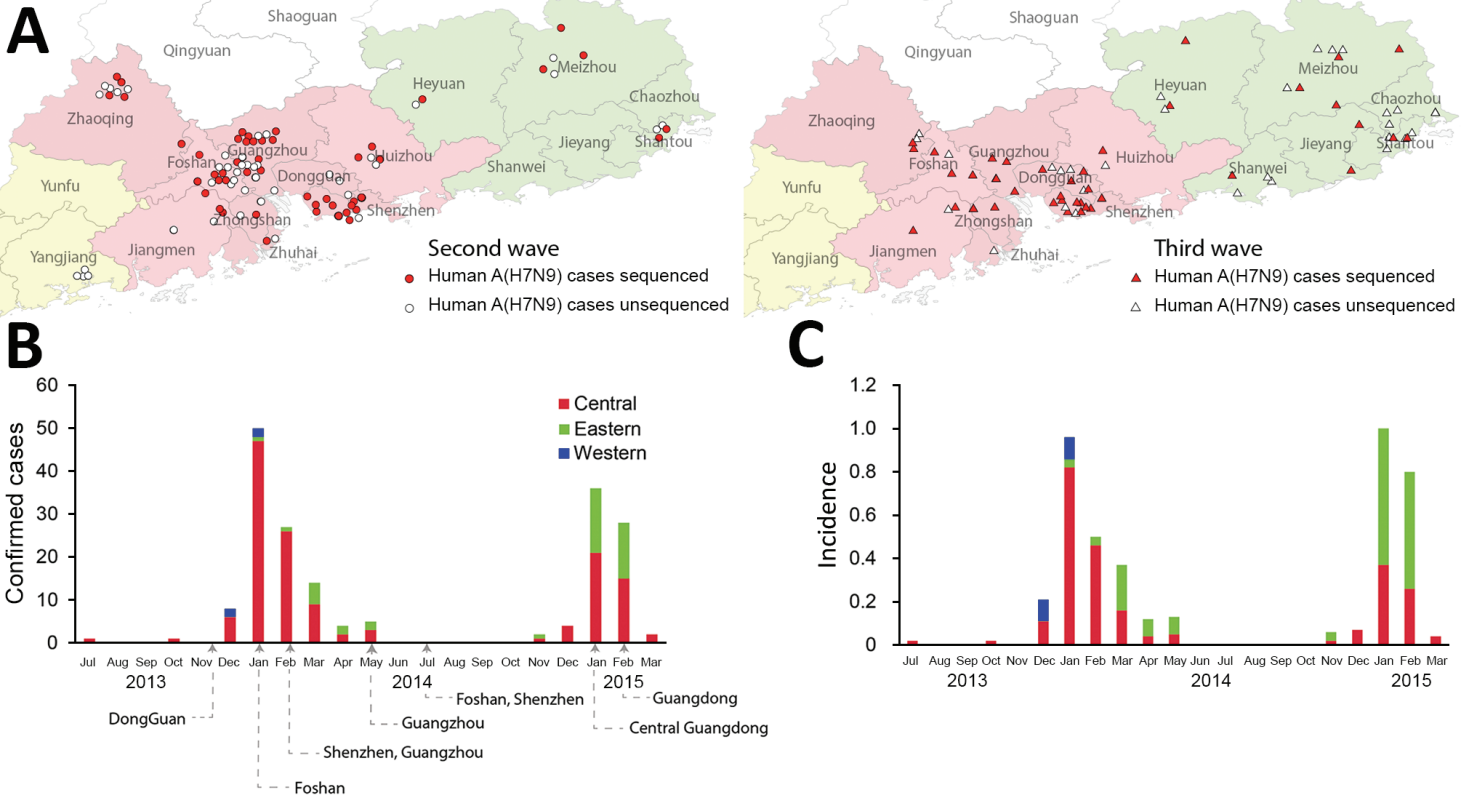
Avian influenza A(H7N9) infection in humans, Guangdong, China, 2013–2015. A) Geographic distribution of H7N9 in humans during the second (June 2013–May 2014) and third (June 2014–May 2015) waves. Confirmed cases in humans identified during the second wave are marked with circles and during the third wave with triangles. H7N9 isolates newly sequenced in this study are highlighted in red. Pink and green shading indicates city prefectures in central and eastern Guangdong Province, respectively. B) Numbers of human H7N9 infections in different regions of Guangdong Province during 2013–2015. Arrows indicate the dates at which live-poultry markets were closed in epicenter cities. No infections were reported during April–October. C) Incidence (human H7N9 infections/1 million population) in each region.

We used a Bayesian discrete phylogeographic approach to investigate spatial dynamics among 9 geographic regions. Specifically, we considered viral movement across eastern China (Shanghai, Zhejiang, Jiangsu, and Shandong provinces); central China (Jiangxi and Hunan provinces); northern China (Beijing, Henan, Hebei, and Xinjiang provinces); southeastern China (Fujian Province); central Guangdong Province (Guangzhou, Huizhou, Foshan, Dongguan, Zhongshan, Shenzhen, Jiangmen, and Zhaoqing [Figure 1]); eastern Guangdong Province (Meizhou, Heyuan, Chaozhou, Jieyang, Shantou, and Shanwei); western Guangdong Province (Yangjiang, Maoming, and Yunfu); and other regions (related sequences isolated from other countries before the H7N9 epidemic). To trace the origin of H7N9 infection, we considered sporadic clinical infection cases from Malaysia and Taiwan as a separate discrete location. Hong Kong adjoins central Guangdong, and most imported live poultry in Hong Kong is from central Guangdong (Zhuhai and Shenzhen). Therefore, Hong Kong and central Guangdong were considered as a single spatial unit. To provide a more realistic reconstruction that includes the directionality of virus transmission, we used an asymmetric continuous-time Markov chain model (*20*) to estimate ancestral locations and location posterior probabilities for each node in the dated phylogenies. Finally, we used TreeAnnotator to obtain maximum clade credibility trees for each gene, which were visualized by using FigTree version 1.4.2 (http://tree.bio.ed.ac.uk). Nucleotide sequences generated in this study were submitted to GISAID (Global Initiative on Sharing All Influenza Data, http://www.gisaid.org) under accession nos. EPI655863–EPI656506 and EPI654015–EPI654495.

## Results

### An Epicenter Shift of A(H7N9) in Humans, Guangdong, 2013–2015

As of October 14, 2015, a total of 182 laboratory-confirmed clinical cases of A(H7N9) infection in humans and 68 deaths were reported in Guangdong (Figure 1; Technical Appendix Table 1). Guangdong reported no clinical A(H7N9) cases during the first epidemic wave (March 2013–May 2013) but had the highest number of cases during the second (110 cases during June 2013–May 2014) and third (72 cases during June 2014–May 2015) epidemic waves.

Unexpectedly, the geographic distributions of A(H7N9) cases in Guangdong differed during the second and third waves (Figure 1, panels B, C). During the second wave, 110 cases were reported from 14 prefecture-level cities in Guangdong. The epicenter of the outbreak was in central Guangdong in the Pearl River Delta (PRD) region (95 [86%] cases). The highest numbers of human cases were reported from cities in Guangdong that had the most LPMs and the highest population density, such as Guangzhou, Shenzhen, and Foshan (Figure 1, panel A). Citywide LPM closures of 2 weeks’ duration were implemented in Guangzhou and Shenzhen cities in February 2014, during the middle of the second wave, and were extended to all prefecture-level cities in central Guangdong in January 2015 during the middle of the third wave (Figure 1, panel B; online Technical Appendix Table 2). All live poultry were removed, and LPM were disinfected once; the markets were cleaned thoroughly with 0.05%–0.1% diluted chlorine solution after poultry removal. Short-term surveillance showed that the A(H7N9) detection rate decreased from 14.83% (112/755) before LPM closure to 1.67% (5/300) on the day when markets reopened, across 31 sampled markets during the second wave (*4*). Another study found that avian influenza virus contamination in LPMs dropped precipitously after cleaning and disinfecting (*21*). Our clinical surveillance data showed that human cases reduced by 55% (Technical Appendix Table 1) in central Guangdong during the third wave but rose in other regions of Guangdong; eastern Guangdong became a new epicenter of the outbreak. Twenty-eight human cases occurred in eastern Guangdong during the first 2 months of 2015, compared with only 2 during the same period in 2014 (Figure 1). The geographic shift of A(H7N9) infection between the second and third waves became more apparent when incidence per capita was measured (Figure 1, panel C) because eastern Guangdong has a lower average population density than the PRD region.

### Epidemic Origins of Human A(H7N9) Infections in Guangdong

We undertook a phylogenetic molecular clock analysis to identify the epidemic origins and transmission dynamics of circulating avian influenza A strains during the third wave. The H7N9 sequences included in our analyses represented 55% (60/110) and 49% (35/72) of all diagnosed H7N9 cases in Guangdong during the second and third waves, respectively. H7N9 and H9N2 viruses obtained from poultry and LPM environmental samples during 2013–2015 also were sequenced, resulting in 44 complete and 79 incomplete virus genomes generated using high-throughput sequencing (*8*,*9*).

**Figure 2:**
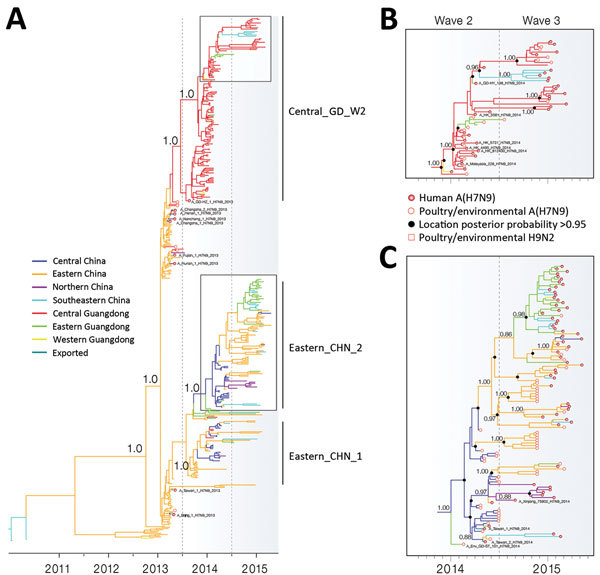
A) Bayesian maximum clade credibility molecular clock tree of influenza virus H7 gene sequences. Branch colors represent the most probable ancestral locations of each branch, inferred from using a spatial phylogenetic model (see Materials and Methods for details). Three major clades of avian influenza A(H7N9) virus were nominated, and phylogenetic posterior probability support is shown for selected clades. B, C) Phylogenies showing hemagglutinin, Guangdong, China, third-wave clades. Phylogenetic posterior probability support is shown for selected clades.

Phylogeographic analysis of 433 H7 HA genes revealed spatial patterns of A(H7N9) transmission across China (Figure 2, panel A). During the first epidemic wave, A(H7N9) virus spread from eastern China to northern, central, and southeastern China, causing a few human infections (Figure 2, panel A), consistent with previous analyses (*22*,*23*). All A(H7N9) viruses identified from chicken and environmental samples in Guangdong during the first wave were derived from viruses circulating in eastern China (Figure 2, panel A). These Guangdong viruses fell into 2 distinct phylogenetic clusters, indicating multiple independent introductions to the region, possibly through different poultry trade routes.

**Figure 3:**
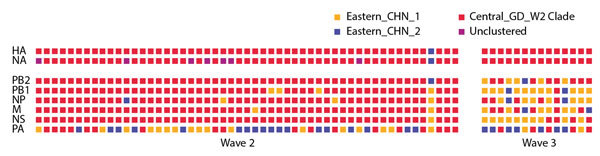
A) Bayesian maximum clade credibility phylogeographic tree of influenza virus polymerase protein 2 gene sequences. Branch colors represent the most probable ancestral locations of each branch, inferred from using a spatial phylogenetic model (see Materials and Methods for details). B–E) Phylogenies showing selected PB2 Guangdong third-wave clades. Empty squares indicate A(H9N2) virus sequences.

During the second epidemic wave, local transmission and proliferation of A(H7N9) virus was detected in Guangdong, particularly in central Guangdong. The HA phylogeny indicated that 94% of A(H7N9) from clinical cases and 92% of H7 subtypes from LPM environmental samples in Guangdong during the second wave clustered into 1 large clade, designated here as Central_GD_W2 (Figure 2, panel A; Figure 3). The earliest second wave case in this clade was sampled in August 2013 (A_GD-HZ_1_H7N9_2013; Figure 2, panel A). Our analysis suggested that viruses related to A_GD-HZ_1_H7N9_2013 persisted in and disseminated through central Guangdong, giving rise to a large number of A(H7N9) cases in the region during the second wave. Moreover, we found that Central_GD_W2 clade viruses also disseminated from central to eastern and western Guangdong during the second wave and caused human cases (e.g., A_GD-HY_138_2014) (Figure 2, panels A, B). Four isolates from humans in Hong Kong and 1 from a human in Malaysia during the second wave also fell within this clade (Figure 2, panel B). The phylogeny of N9 NA sequences displayed similar virus transmission patterns (online Technical Appendix Figure, panel A). Most (73%) N9 genes from A(H7N9) isolates in Guangdong during the second wave clustered within the Central_GD_W2 clade. In contrast to HA, the NA phylogeny showed that A(H7N9) isolates during the second wave in central Guangdong did not form a single cluster (Technical Appendix Figure, panel A). However, fewer human cases (27%) were caused by these viruses, and most cases were limited to Shenzhen and Shantou cities, suggesting less transmission of viruses in this clade (Technical Appendix Figure, panel A).

The incidence of A(H7N9) in humans in central Guangdong decreased by 55% during the third wave (Figure 1, panel B; Technical Appendix Table 1). However, phylogenetic analysis indicated that the virus persisted in central Guangdong. The H7 phylogeny showed that all A(H7N9) viruses from central Guangdong during the third wave were descended from Central_GD_W2 clade viruses of the second wave (Figure 2, panel B). The outbreaks of A(H7N9) in humans in Fujian Province (southeastern China) during the third wave also were caused by Central_GD_W2 clade viruses, suggesting a possible transmission of A(H7N9) virus from central Guangdong to cities in southeastern China during the second wave.

A major feature during the third epidemic wave was an increase in A(H7N9) cases in humans in eastern Guangdong (Figure 1, panels B, C; Technical Appendix Table 1). Most (94%) isolates identified in eastern Guangdong during the third wave clustered into a single subclade of Eastern_CHN_W2 clade viruses in both the H7 and N9 phylogenies (Figure 2, panel C; Technical Appendix Figure, panel A). The Eastern_CHN_W2 clade viruses were mainly found in poultry from central China during the second wave but became predominant (among both poultry and humans) in eastern China and eastern Guangdong during the third wave (Figure 2, panel C). Moreover, these viruses formed location-specific clades during the third wave, suggesting that they have become established and enzootic to the poultry populations in each locality (Figure 2, panel C).

### Genetic Diversities of A(H7N9) Virus in Guangdong

**Figure 4:**
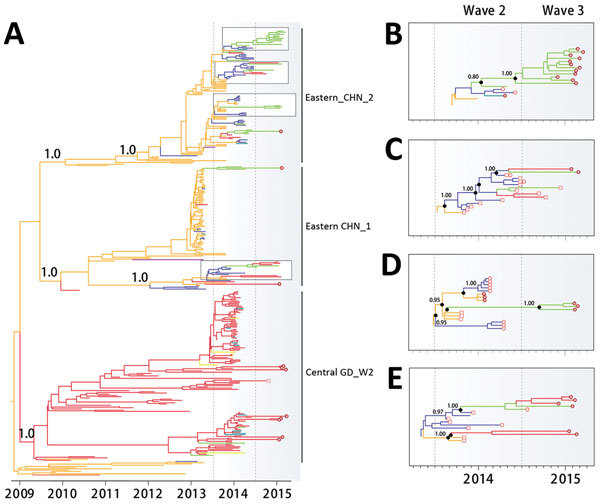
Genotypic analysis of influenza A(H7N9) viruses. Proposed genotypes are shown for 68 fully sequenced A(H7N9) viruses isolated from humans in central Guangdong during 2013–2015. Each square represents a gene sequence, and its color indicates the most probable clade to which that sequence belongs, as inferred from the phylogenies in Figures 2 and 3 and online Technical Appendix Figure, panels B–F (http://wwwnc.cdc.gov/EID/article/22/12/16-0450-Techapp1.pdf).

During the second wave, sequences from 4 internal genes (NP, NS, PB1, PB2) of A(H7N9) isolates from central Guangdong mostly clustered into a single major clade, together with local A(H9N2) strains; these sequences were distinct from the A(H7N9) sequences from central or eastern China (Figure 4, panel A; Technical Appendix Figure, panels B, D, F) (*8*,*22*). However, by the third wave, most A(H7N9) viruses from central Guangdong had acquired some internal genes from viruses from central or eastern China. In particular, PB1, PB2, NP, and NS sequences obtained from humans with A(H7N9) infection and from H7N9/H9N2 environmental samples in central Guangdong during the third wave were distinct from those from the second wave (Figure 4, panel A; Technical Appendix Figure, panels B, D, F). For instance, analysis of the PB2 gene revealed that 65 of 69 viruses from clinical samples clustered into the Central_GD_W2 clade during the second wave. However, during the third wave, only 8 of 32 H7N9 viruses from clinical isolates fell into this group. The remaining 24 H7N9 isolates were phylogenetically related to H7N9 or H9N2 isolates from central or eastern China. It therefore appears that >70% of third-wave H7N9 viruses from central Guangdong acquired a PB2 gene through reassortment with strains circulating elsewhere. A similar pattern also was observed for H7N9 and H9N2 isolates from LPM environments (Figure 4). These findings supported the hypothesis that H9N2 viruses previously circulating outside Guangdong were introduced into central Guangdong during the third wave and increased the diversity of internal genes of H7N9 in this place by reassortment.

We further classified all human A(H7N9) isolates from central Guangdong according to the phylogenetic placement of their genome segments. Genome segments of second-wave A(H7N9) from central Guangdong were typically co-inherited (except polymerase) and belonged to the Central_GD_W2 clade (Figure 3; Figure 4, panel A; Technical Appendix Figure). A total of 12 different lineages were observed in 55 human A(H7N9) viruses isolated during the second wave in central Guangdong. This pattern was absent during the third wave; instead, the genomic structures of human A(H7N9) viruses were highly variable, with the exception of the HA and NA segments, which retained their association with the Central_GD_W2 clade (Figure 3).

In contrast, phylogenetic reconstruction of PB1, PB2, NP, and NS sequences indicated that most third-wave sequences seen in eastern Guangdong were genetically similar (Figure 4, panel B; Technical Appendix Figure, panels. B–F). These sequences grouped into a single clade, whereas those sampled in central Guangdong were phylogenetically dispersed.

## Discussion

We analyzed epidemiologic data pertaining to influenza A(H7N9) virus in humans and virus sequence data from 52% (95/182) of all persons in whom A(H7N9) infection was diagnosed and from LPMs environment samples during 2013–2015 to characterize the origin and transmission of A(H7N9) in humans across epidemic waves in Guangdong. The phylogeographic analyses of HA and NA indicate that the virus strain that caused third-wave outbreaks in central Guangdong descend from second-wave viruses that circulated in the same region. In other words, H7N9 circulating during the second wave probably persisted in targeted cities and/or their neighboring areas until the third epidemic wave.

In contrast, the histories of the H7N9 internal genes, which are affected by frequent reassortment with prevalent H9N2 viruses, most likely mirror the trade routes of live poultry. We found that A(H7N9) viruses from central Guangdong during the second wave possessed 5 (PB2, PB1, matrix, NP, NS) of 6 internal genes mainly from the Central_GD_W2 clade. However, in the third wave, viral internal genes were dispersed in phylogenetic trees; most strains fell into the Eastern_CHN1 or Eastern_CHN2 clades, suggesting another source for the parental viruses of these reassortants (Figure 3). This finding suggests there were changes in nature of A(H9N2) viruses that were co-circulating in central Guangdong (Figure 4, panel C; Technical Appendix Figures B–F). Our data indicate that the genetic diversity of H7N9 or H9N2 in central Guangdong increased during the third wave, even though the number of human infections was lower in this region.

From a public health standpoint, our results underscore 3 major concerns with current A(H7N9) infections in humans in China. First, the persistence and spread of A(H7N9) have not been completely constrained. A(H7N9) circulating in central Guangdong during the second wave was persistent and caused outbreaks in humans during the third wave. For example, 5 and 3 cases, respectively, were identified in humans in March 2014 in Shenzhen and Guangzhou in Guangdong after the reopening of LPMs in February 2014 (Technical Appendix Tables 1, 2). This finding suggests that interventions such as the temporary closure and sanitation of LPMs can reduce virus contamination in poultry and environmental samples but apparently cannot eliminate the risk for human infection (*21*). Recent long-term LPM surveillance in Guangzhou suggests that different types of poultry markets were recontaminated by A(H7N9) and other avian influenza A strains; up to 2 days after markets were reopened, detection rates of the viruses in LPM environments were as high as those before market closure (*5*).

Second, a geographic shift of the epicenter of human infections between the second and third waves in Guangdong suggests an epidemic of A(H7N9) infection is difficult to control solely by interfering in the epicenter of an outbreak. Some regions, once contaminated, might act as sources of infection to the wider poultry sector. Current sequence data suggest that the A(H7N9) human infections in eastern, northern, and southeastern China and eastern Guangdong during the third wave were mainly caused by viruses belonging to the Eastern_CHN_W2 clade, which were predominantly isolated from poultry populations in central China at the end of the second wave (Figure 2, panel C). In eastern Guangdong, 94% of A(H7N9) cases in humans during the third wave were caused by Eastern_CHN_W2 clade viruses.

Third, other measures complementary to LPM closures should be considered by government administration. We observed a continued reassortment of Guangdong A(H7N9) lineages with viral strains from central and eastern China since February 2014 (Figures 3, 4), suggesting that live poultry from central and eastern areas might have been introduced into central Guangdong after the local LPMs were closed. Indeed, it is plausible that official closure led to an increase in illicit trading in some markets or neighborhoods (http://gzdaily.dayoo.com/html/2015-03/18/content_2885023.htm; http://shenzhen.sina.com.cn/news/n/2015-04-20/detail-iavxeafs5854802.shtml). In this context, the closure of central LPMs without a strict ban on the live poultry trade could, at least in theory, have detrimental effects. Illicit trading has the potential to change the poultry trade and make officially monitoring and controlling it more difficult. Other less disruptive measures that have been proven to reduce risk can be considered, such as rest days and banning live poultry overnight (*24*).

One limitation of this study is a lack of long-term surveillance of live poultry in Guangdong. Such surveillance is difficult to implement because of a low infection rate and the absence of signs during A(H7N9) infection in poultry. However, most A(H7N9) infection in humans results from direct exposure to live poultry (*25*) (Technical Appendix Table 3). Furthermore, we used in our analyses environmental samples from LPMs, which partially reflect the circulation of avian influenza A strains in poultry. In addition, surveillance efforts in different regions might differ and could affect our interpretation on virus origin and transmission. In this study, we have analyzed most publicly available, genetically related A(H7N9) sequences out of Guangdong Province, and the regions of eastern China and central Guangdong that reported most clinical H7N9 cases represent most H7N9-related sequences. However, sampling bias cannot be excluded, especially for environmental and poultry samples. Central China is a major poultry farming area but has a limited number of A(H7N9) sequences from poultry, which could be caused by a lower prevalence of the virus in this region or by a possible limited surveillance effort in poultry population. As a result, sequences data from Eastern_CHN_W2 clade are still limited to illustrate the evolution and transmission route of this clade of A(H7N9) (Figure 2, panel C). Longer-term and larger-scale studies are necessary to provide more robust evidence about the value of interventions for controlling the epidemic at national and regional scales.

During February 5–11, 2016, twenty-eight new A(H7N9)cases were reported in humans. Clearly, the public health risk from A(H7N9) remains. Our results highlight some limitations of the current geographically restricted LPM interventions on the epidemic control of A(H7N9), which might also apply to other avian influenza viruses. To focus only on end-stage epidemic LPM interventions without including the entirety of the LPM transmission chain might, however, be of limited value. Public health organizations might wish to consider the possibility of proactively closing LPMs, and alternative measures as recently suggested (*24*), in areas potentially at-risk, although we recognize that practical, economic, and administrative considerations also contribute to decision-making processes.

Technical Appendix Further details on investigation of the effects of live-poultry market interventions on avian influenza A(H7N9) virus in humans, Guangdong, March 2013–October 2015.
