# Supplementary material for: Effect of Live Poultry Market Interventions on Influenza A(H7N9) Virus, Guangdong, China
Source: Emerg Infect Dis. 2016 Dec;22(12):2104–12. doi: 10.3201/eid2212.160450 (PMC5189139; doi:10.3201/eid2212.160450)
Supplement: Technical Appendix — Further details on investigation of the effects of live-poultry market interventions on avian influenza A(H7N9) virus in humans, Guangdong, March 2013–October 2015. [file 16-0450-Techapp-s1.pdf]

Article DOI: http://dx.doi.org/10.3201/eid2212.160450

# Live Poultry Market Interventions for Influenza A (H7N9) Virus, Guangdong, China

## Technical Appendix

**Technical Appendix Table 1.** Reported avian influenza A(H7N9) cases in humans, Guangdong Province, China, March 2013–October 2015

| | Wave 2, 2013 Jun–2014 May | | | | | | | | Wave 3, 2014 Jun–2015 May | | | | |
|---|---|---|---|---|---|---|---|---|---|---|---|---|---|
| Prefectual cities | Jul | Oct | Dec | Jan | Feb | Mar | Apr | May | Nov | Dec | Jan | Feb | Mar |
| Dongguan (DG)* | | 1 | 1 | 1 | | | | | 1 | 2 | 3 | 2 | |
| Foshan (FS)* | | | 3 | 8 | 4 | | | | | | 1 | 2 | 1 |
| Guangzhou (GZ)* | | | | 7 | 14 | 3 | 1 | | | | 1 | 4 | |
| Huizhou (HZ)* | 1 | | | 3 | | 1 | | | | | 1 | 1 | |
| Jiangmen (JM)* | | | | 3 | 2 | | | 1 | | | 0 | 2 | 1 |
| Shenzhen (SZ)* | | | 2 | 16 | 1 | 5 | 1 | | | 1 | 11 | 1 | |
| Zhuhai (ZH)* | | | | | 1 | | | | | | 1 | | |
| Zhaoqing (ZQ)* | | | | 6 | 4 | | | | | 1 | 1 | 2 | |
| Zhongshan (ZS)* | | | | 3 | | | | 2 | | | 2 | 1 | |
| Central Guangdong | | | | 95 | | | | | | | 43 | | |
| Chaozhou (CZ)† | | | | | | 1 | | | | | 2 | 3 | |
| Heyuan (HY)† | | | | | | 2 | | | | | 2 | 2 | |
| Jieyang (JY)† | | | | | | | | | | | 2 | | |
| Meizhou (MZ)† | | | | 1 | 1 | 1 | | 2 | 1 | | 4 | 3 | |
| Shantou (ST)† | | | | | | 1 | 2 | | | | 1 | 5 | |
| Shanwei (SW)† | | | | | | | | | | | 4 | | |
| Eastern Guangdong | | | | 11 | | | | | | | 29 | | |
| Yangjiang (YJ) | | | 2 | 2 | | | | | | | 0 | | |
| Guangdong | | | | 110 | | | | | | | 72 | | |

* Cities in Central Guangdong.

† Cities in Eastern Guangdong.

**Technical Appendix Table 2.** Interventions implemented in LPMs during epidemics of avian influenza A(H7N9) virus, Guangdong Province, China*

| City | Time | LPM interventions | Reference |
| --- | --- | --- | --- |
| Dongguan | 2013 Nov–2014 Jan | Ten LPMs of the city were separately closed for 2 wks where human infection cases were identified | Kang, et al.† |
| Foshan | 2014 Jan 13–2014 Jan 29 | LPMs of Nanhai and Chancheng districts were closed | Wu et al.‡ |
| Shenzhen | 2014 Jan 31–2014 Feb 13 | All LPMs of the city were closed; cleaning and disinfecting were conducted | Wu et al.‡ |
| Guangzhou | 2014 Feb 15–2014 Feb 28 | All LPMs of the city were closed; cleaning and disinfecting were conducted | Wu, et al.‡ |
| Guangzhou | 2014 May 5–current | Four districts of Guangzhou ban the sales of live poultry, and only frozen poultry was allowed | http://www.chinadailyasia.com/news/2014-04/30/content_15132977.html |
| Foshan | 2014 July 1–current | Chancheng district ban the sales of live poultry, and only frozen poultry was allowed | http://gzdaily.dayoo.com/html/2014-06/30/content_2674385.htm (In Chinese) |
| Shenzhen | 2014 July 1–current | Futian district ban the sales of live poultry, and only frozen poultry was allowed | http://gzdaily.dayoo.com/html/2014-06/30/content_2674385.htm (In Chinese) |
| Central Guangdong | 2015 Jan 15 | Cities at various levels in the Pear River Delta were required to ban the sale of live poultry | http://www.wantchinatimes.com/news-subclass-cnt.aspx?id=20150126000004&cid=1201 |
| Guangzhou | 2015 Feb 13–2015 Feb 18 | All LPMs of the city were closed; cleaning and disinfecting were conducted | http://www.chinanews.com/tp/2015/02-13/7063641.shtml |
| Guangdong Province | 2015 Feb 19–2015 Feb.28 | All LPMs were closed; cleaning and disinfecting were conducted | http://news.sina.com.cn/o/2015-02-15/193231524882.shtml (In Chinese) |

*LPM, live-poultry market.

†Kang M, He J, Song T, Rutherford S, Wu J, Lin J, et al. Environmental sampling for avian influenza A(H7N9) in live-poultry markets in Guangdong, China. PLoS One. 2015;10:e0126335.

‡Wu P, Jiang H, Wu JT, Chen E, He J, Zhou H, et al. Poultry market closures and human infection with influenza A(H7N9) virus, China, 2013–14. Emerg Infect Dis. 2014 Nov;20:1891–4.

**Technical Appendix Table 3.** Epidemiologic characteristics of 182 patients with confirmed avian influenza A(H7N9)virus infection, Guangdong, China, 2013–2015

| | Patients with confirmed cases | |
|---|---|---|
| Characteristic | Wave 2, n = 110 | Wave 3, n = 72 |
| Age, y | | |
| Median | 55.5 | 52 |
| Interquartile range | 3–88 | 0.8–83 |
| Male sex, no. (%) | 68 (61.8) | 51 (70.8) |
| Type of residence, no. (%) | | |
| Rural | 21 (19) | 13 (18) |
| Urban | 79 (81) | 59 (82) |
| A(H7N9) cluster | 3 | 3* |
| No. deaths (%) | 37 (33.6) | 31 (43.1) |

*A(H7N9) infection was diagnosed in 2 physicians in a respiratory unit at a hospital in Shantou city, Guangdong, with no known recent exposure to live poultry.

**Technical Appendix Table 4.** Global Initiative on Sharing All Influenza Data accession numbers of avian influenza A(H7N9) viruses isolated from humans in a study of the effect of live-poultry market interventions, Guangdong, China*

| A(H7N9) clinical strains† | HA | NA | PB2 | PB1 | PA | NP | M | NS |
|---|---|---|---|---|---|---|---|---|
| A/GD-10/2014/H7N9/2014-01-03 | EPI655870 | EPI655869 | EPI655867 | EPI655868 | EPI655866 | EPI655863 | EPI655865 | EPI655864 |
| A/GD-12/2014/H7N9/2014-01-03 | EPI655878 | EPI655877 | EPI655875 | EPI655876 | EPI655874 | EPI655871 | EPI655873 | EPI655872 |
| A/GD-13/2014/H7N9/2014-01-03 | EPI655886 | EPI655885 | EPI655883 | EPI655884 | EPI655882 | EPI655879 | EPI655881 | EPI655880 |
| A/GD-18/2014/H7N9/2014-01-07 | EPI655894 | EPI655893 | EPI655891 | EPI655892 | EPI655890 | EPI655887 | EPI655889 | EPI655888 |
| A/GD-19/2014/H7N9/2014-01-07 | EPI655902 | EPI655901 | EPI655899 | EPI655900 | EPI655898 | EPI655895 | EPI655897 | EPI655896 |
| A/GD-24/2014/H7N9/2014-01-10 | EPI655910 | EPI655909 | EPI655907 | EPI655908 | EPI655906 | EPI655903 | EPI655905 | EPI655904 |
| A/GD-26/2014/H7N9/2014-01-10 | EPI655918 | EPI655917 | EPI655915 | EPI655916 | EPI655914 | EPI655911 | EPI655913 | EPI655912 |
| A/GD-29/2014/H7N9/2014-01-11 | EPI655926 | EPI655925 | EPI655923 | EPI655924 | EPI655922 | EPI655919 | EPI655921 | EPI655920 |
| A/GD-31/2014/H7N9/2014-01-11 | EPI655934 | EPI655933 | EPI655931 | EPI655932 | EPI655930 | EPI655927 | EPI655929 | EPI655928 |
| A/GD-33/2014/H7N9/2014-01-12 | EPI655942 | EPI655941 | EPI655939 | EPI655940 | EPI655938 | EPI655935 | EPI655937 | EPI655936 |
| A/GD-34/2014/H7N9/2014-01-12 | EPI655950 | EPI655949 | EPI655947 | EPI655948 | EPI655946 | EPI655943 | EPI655945 | EPI655944 |
| A/GD-36/2014/H7N9/2014-01-12 | EPI655958 | EPI655957 | EPI655955 | EPI655956 | EPI655954 | EPI655951 | EPI655953 | EPI655952 |
| A/GD-35/2014/H7N9/2014-01-12 | EPI655966 | EPI655965 | EPI655963 | EPI655964 | EPI655962 | EPI655959 | EPI655961 | EPI655960 |
| A/GD-43/2014/H7N9/2014-01-15 | EPI655974 | EPI655973 | EPI655971 | EPI655972 | EPI655970 | EPI655967 | EPI655969 | EPI655968 |
| A/GD-46/2014/H7N9/2014-01-16 | EPI655982 | EPI655981 | EPI655979 | EPI655980 | EPI655978 | EPI655975 | EPI655977 | EPI655976 |
| A/GD-48/2014/H7N9/2014-01-19 | EPI655990 | EPI655989 | EPI655987 | EPI655988 | EPI655986 | EPI655983 | EPI655985 | EPI655984 |
| A/GD-62/2014/H7N9/2014-01-27 | EPI655998 | EPI655997 | EPI655995 | EPI655996 | EPI655994 | EPI655991 | EPI655993 | EPI655992 |
| A/GD-65/2014/H7N9/2014-01-29 | EPI656006 | EPI656005 | EPI656003 | EPI656004 | EPI656002 | EPI655999 | EPI656001 | EPI656000 |
| A/GD-66/2014/H7N9/2014-01-29 | EPI656014 | EPI656013 | EPI656011 | EPI656012 | EPI656010 | EPI656007 | EPI656009 | EPI656008 |
| A/GD-69/2014/H7N9/2014-01-30 | EPI656022 | EPI656021 | EPI656019 | EPI656020 | EPI656018 | EPI656015 | EPI656017 | EPI656016 |
| A/GD-81/2014/H7N9/2014-01-30 | EPI656030 | EPI656029 | EPI656027 | EPI656028 | EPI656026 | EPI656023 | EPI656025 | EPI656024 |
| A/GD-71/2014/H7N9/2014-01-30 | EPI656038 | EPI656037 | EPI656035 | EPI656036 | EPI656034 | EPI656031 | EPI656033 | EPI656032 |
| A/GD-75/2014/H7N9/2014-01-31 | EPI656046 | EPI656045 | EPI656043 | EPI656044 | EPI656042 | EPI656039 | EPI656041 | EPI656040 |
| A/GD-74/2014/H7N9/2014-01-31 | EPI656054 | EPI656053 | EPI656051 | EPI656052 | EPI656050 | EPI656047 | EPI656049 | EPI656048 |
| A/GD-82/2014/H7N9/2014-02-02 | EPI656062 | EPI656061 | EPI656059 | EPI656060 | EPI656058 | EPI656055 | EPI656057 | EPI656056 |

| A(H7N9) clinical strains† | HA | NA | PB2 | PB1 | PA | NP | M | NS |
|---|---|---|---|---|---|---|---|---|
| A/GD-98/2014/H7N9/2014-02-06 | EPI656070 | EPI656069 | EPI656067 | EPI656068 | EPI656066 | EPI656063 | EPI656065 | EPI656064 |
| A/GD-101/2014/H7N9/2014-02-07 | EPI656078 | EPI656077 | EPI656075 | EPI656076 | EPI656074 | EPI656071 | EPI656073 | EPI656072 |
| A/GD-103/2014/H7N9/2014-02-10 | EPI656086 | EPI656085 | EPI656083 | EPI656084 | EPI656082 | EPI656079 | EPI656081 | EPI656080 |
| A/GD-104/2014/H7N9/2014-02-11 | EPI656094 | EPI656093 | EPI656091 | EPI656092 | EPI656090 | EPI656087 | EPI656089 | EPI656088 |
| A/GD-105/2014/H7N9/2014-02-11 | EPI656102 | EPI656101 | EPI656099 | EPI656100 | EPI656098 | EPI656095 | EPI656097 | EPI656096 |
| A/GD-109/2014/H7N9/2014-02-13 | | | EPI656107 | EPI656108 | EPI656106 | EPI656103 | EPI656104 | EPI656105 |
| A/GD-110/2014/H7N9/2014-02-13 | EPI656116 | EPI656115 | EPI656113 | EPI656114 | EPI656112 | EPI656109 | EPI656111 | EPI656110 |
| A/GD-112/2014/H7N9/2014-02-18 | EPI656124 | EPI656123 | EPI656121 | EPI656122 | EPI656120 | EPI656117 | EPI656119 | EPI656118 |
| A/GD-114/2014/H7N9/2014-02-18 | EPI656132 | EPI656131 | EPI656129 | EPI656130 | EPI656128 | EPI656125 | EPI656127 | EPI656126 |
| A/GD-119/2014/H7N9/2014-02-19 | EPI656140 | EPI656139 | EPI656137 | EPI656138 | EPI656136 | EPI656133 | EPI656135 | EPI656134 |
| A/GD-120/2014/H7N9/2014-02-19 | EPI656148 | EPI656147 | EPI656145 | EPI656146 | EPI656144 | EPI656141 | EPI656143 | EPI656142 |
| A/GD-121/2014/H7N9/2014-02-19 | EPI656156 | EPI656155 | EPI656153 | EPI656154 | EPI656152 | EPI656149 | EPI656151 | EPI656150 |
| A/GD-116/2014/H7N9/2014-02-22 | | EPI656163 | EPI656161 | EPI656162 | EPI656160 | EPI656157 | EPI656159 | EPI656158 |
| A/GD-117/2014/H7N9/2014-02-22 | EPI656171 | EPI656170 | EPI656168 | EPI656169 | EPI656167 | EPI656164 | EPI656166 | EPI656165 |
| A/GD-123/2014/H7N9/2014-03-01 | EPI656179 | EPI656178 | EPI656176 | EPI656177 | EPI656175 | EPI656172 | EPI656174 | EPI656173 |
| A/GD-124/2014/H7N9/2014-03-06 | EPI656187 | EPI656186 | EPI656184 | EPI656185 | EPI656183 | EPI656180 | EPI656182 | EPI656181 |
| A/GD-125/2014/H7N9/2014-03-09 | EPI656195 | EPI656194 | EPI656192 | EPI656193 | EPI656191 | EPI656188 | EPI656190 | EPI656189 |
| A/GD-126/2014/H7N9/2014-03-10 | EPI656203 | EPI656202 | EPI656200 | EPI656201 | EPI656199 | EPI656196 | EPI656198 | EPI656197 |
| A/GD-136/2014/H7N9/2014-03-29 | EPI656211 | EPI656210 | EPI656208 | EPI656209 | EPI656207 | EPI656204 | EPI656206 | EPI656205 |
| A/GD-138/2014/H7N9/2014-04-05 | EPI656219 | EPI656218 | EPI656216 | EPI656217 | EPI656215 | EPI656212 | EPI656214 | EPI656213 |
| A/GD-139/2014/H7N9/2014-04-06 | EPI656226 | EPI656221 | EPI656223 | EPI656224 | EPI656222 | EPI656225 | EPI656220 | |
| A/GD-151/2014/H7N9/2014-05-06 | EPI656234 | EPI656233 | EPI656231 | EPI656232 | EPI656230 | EPI656227 | EPI656229 | EPI656228 |
| A/GD-153/2014/H7N9/2014-05-08 | EPI656242 | EPI656241 | EPI656239 | EPI656240 | EPI656238 | EPI656235 | EPI656237 | EPI656236 |
| A/GD-154/2014/H7N9/2014-05-28 | EPI656250 | EPI656249 | EPI656247 | EPI656248 | EPI656246 | EPI656243 | EPI656245 | EPI656244 |
| A/GD-155/2014/H7N9/2014-11-19 | EPI656258 | EPI656257 | EPI656255 | EPI656256 | EPI656254 | EPI656251 | EPI656253 | EPI656252 |
| A/GD-156/2014/H7N9/2014-11-25 | EPI656266 | EPI656265 | EPI656263 | EPI656264 | EPI656262 | EPI656259 | EPI656261 | EPI656260 |
| A/GD-1/2015/H7N9/2015-01-03 | EPI656274 | EPI656273 | EPI656271 | EPI656272 | EPI656270 | EPI656267 | EPI656269 | EPI656268 |

| A(H7N9) clinical strains† | HA | NA | PB2 | PB1 | PA | NP | M | NS |
|---|---|---|---|---|---|---|---|---|
| A/GD-2/2015/H7N9/2015-01-06 | EPI656282 | EPI656281 | EPI656279 | EPI656280 | EPI656278 | EPI656275 | EPI656277 | EPI656276 |
| A/GD-10/2015/H7N9/2015-01-06 | EPI656290 | EPI656289 | EPI656287 | EPI656288 | EPI656286 | EPI656283 | EPI656285 | EPI656284 |
| A/GD-18/2015/H7N9/2015-01-11 | EPI656298 | EPI656297 | EPI656295 | EPI656296 | EPI656294 | EPI656291 | EPI656293 | EPI656292 |
| A/GD-17/2015/H7N9/2015-01-11 | EPI656306 | EPI656305 | EPI656303 | EPI656304 | EPI656302 | EPI656299 | EPI656301 | EPI656300 |
| A/GD-20/2015/H7N9/2015-01-15 | EPI656314 | EPI656313 | EPI656311 | EPI656312 | EPI656310 | EPI656307 | EPI656309 | EPI656308 |
| A/GD-21/2015/H7N9/2015-01-16 | EPI656322 | EPI656321 | EPI656319 | EPI656320 | EPI656318 | EPI656315 | EPI656317 | EPI656316 |
| A/GD-44/2015/H7N9/2015-01-19 | EPI656330 | EPI656329 | EPI656327 | EPI656328 | EPI656326 | EPI656323 | EPI656325 | EPI656324 |
| A/GD-27/2015/H7N9/2015-01-21 | EPI656338 | EPI656337 | EPI656335 | EPI656336 | EPI656334 | EPI656331 | EPI656333 | EPI656332 |
| A/GD-43/2015/H7N9/2015-01-22 | EPI656346 | EPI656345 | EPI656343 | EPI656344 | EPI656342 | EPI656339 | EPI656341 | EPI656340 |
| A/GD-30/2015/H7N9/2015-01-22 | EPI656354 | EPI656353 | EPI656351 | EPI656352 | EPI656350 | EPI656347 | EPI656349 | EPI656348 |
| A/GD-42/2015/H7N9/2015-01-25 | EPI656362 | EPI656361 | EPI656359 | EPI656360 | EPI656358 | EPI656355 | EPI656357 | EPI656356 |
| A/GD-45/2015/H7N9/2015-01-26 | EPI656370 | EPI656369 | EPI656367 | EPI656368 | EPI656366 | EPI656363 | EPI656365 | EPI656364 |
| A/GD-54/2015/H7N9/2015-01-27 | EPI656378 | EPI656377 | EPI656375 | EPI656376 | EPI656374 | EPI656371 | EPI656373 | EPI656372 |
| A/GD-55/2015/H7N9/2015-01-27 | EPI656386 | EPI656385 | EPI656383 | EPI656384 | EPI656382 | EPI656379 | EPI656381 | EPI656380 |
| A/GD-50/2015/H7N9/2015-01-27 | EPI656394 | EPI656393 | EPI656391 | EPI656392 | EPI656390 | EPI656387 | EPI656389 | EPI656388 |
| A/GD-51/2015/H7N9/2015-01-30 | EPI656402 | EPI656401 | EPI656399 | EPI656400 | EPI656398 | EPI656395 | EPI656397 | EPI656396 |
| A/GD-53/2015/H7N9/2015-02-03 | EPI656410 | EPI656409 | EPI656407 | EPI656408 | EPI656406 | EPI656403 | EPI656405 | EPI656404 |
| A/GD-57/2015/H7N9/2015-02-03 | EPI656418 | EPI656417 | EPI656415 | EPI656416 | EPI656414 | EPI656411 | EPI656413 | EPI656412 |
| A/GD-69/2015/H7N9/2015-02-04 | EPI656426 | EPI656425 | EPI656423 | EPI656424 | EPI656422 | EPI656419 | EPI656421 | EPI656420 |
| A/GD-72/2015/H7N9/2015-02-07 | EPI656434 | EPI656433 | EPI656431 | EPI656432 | EPI656430 | EPI656427 | EPI656429 | EPI656428 |
| A/GD-68/2015/H7N9/2015-02-08 | EPI656442 | EPI656441 | EPI656439 | EPI656440 | EPI656438 | EPI656435 | EPI656437 | EPI656436 |
| A/GD-80/2015/H7N9/2015-02-10 | EPI656450 | EPI656449 | EPI656447 | EPI656448 | EPI656446 | EPI656443 | EPI656445 | EPI656444 |
| A/GD-81/2015/H7N9/2015-02-10 | EPI656458 | EPI656457 | EPI656455 | EPI656456 | EPI656454 | EPI656451 | EPI656453 | EPI656452 |
| A/GD-76/2015/H7N9/2015-02-10 | EPI656466 | EPI656465 | EPI656463 | EPI656464 | EPI656462 | EPI656459 | EPI656461 | EPI656460 |
| A/GD-92/2015/H7N9/2015-02-26 | EPI656474 | EPI656473 | EPI656471 | EPI656472 | EPI656470 | EPI656467 | EPI656469 | EPI656468 |
| A/GD-91/2015/H7N9/2015-02-26 | EPI656482 | EPI656481 | EPI656479 | EPI656480 | EPI656478 | EPI656475 | EPI656477 | EPI656476 |
| A/GD-124/2015/H7N9/2015-02-26 | EPI656490 | EPI656489 | EPI656487 | EPI656488 | EPI656486 | EPI656483 | EPI656485 | EPI656484 |

| A(H7N9) clinical strains† | HA | NA | PB2 | PB1 | PA | NP | M | NS |
|---|---|---|---|---|---|---|---|---|
| A/GD-95/2015/H7N9/2015-03-05 | EPI656498 | EPI656497 | EPI656495 | EPI656496 | EPI656494 | EPI656491 | EPI656493 | EPI656492 |
| A/GD-120/2015/H7N9/2015-03-10 | EPI656506 | EPI656505 | EPI656503 | EPI656504 | EPI656502 | EPI656499 | EPI656501 | EPI656500 |

*HA, hemagglutinin; M, matrix; NA, neuraminidase; NP, nucleoprotein; NS, nonstructural; PA, polymerase; PB, polymerase protein.

†Accession number for H7N9 and H9N2 strains from live-poultry market environment are EPI654015–EPI654495.

**Technical Appendix Table 5.** Geographic distribution of avian influenza A(H7N9) viruses sequences used in phylogeographic analyses*

| Gene segments | China | | | | Guangdong | | | | Other countries | Exported regions |
|---|---|---|---|---|---|---|---|---|---|---|
| | Central | Eastern | Southeastern | Northern | Central | Eastern | Western | Hong Kong | | |
| HA | 42 | 163 | 24 | 13 | 135 | 36 | 7 | 9 | – | 4 |
| NA | 42 | 129 | 23 | 15 | 116 | 35 | 15 | 12 | 5 | 4 |
| PB2 | 47 | 130 | 2 | 3 | 109 | 34 | 8 | 35 | – | 3 |
| PB1 | 39 | 107 | 2 | 7 | 104 | 33 | 7 | 19 | 3 | 3 |
| PA | 32 | 127 | 2 | 9 | 125 | 29 | 10 | 27 | 9 | 4 |
| M | 49 | 149 | 2 | 6 | 132 | 38 | 13 | 22 | 6 | 4 |
| NP | 54 | 128 | 3 | 11 | 121 | 33 | 11 | 15 | 7 | 3 |
| NS | 37 | 104 | 1 | 8 | 118 | 33 | 10 | 21 | 11 | 3 |

*HA, hemagglutinin; M, matrix; NA, neuraminidase; NP, nucleoprotein; NS, nonstructural; PA, polymerase; PB, polymerase protein.

**Technical Appendix Figure (following pages).** Bayesian maximum clade credibility phylogeographic tree of the neuraminidase (A), polymerase protein 1 (B), polymerase (C), matrix (D), nucleoprotein (E), and nonstructural (F) gene sequences. Branch colors represent the most probable ancestral locations inferred from geo-referenced sequence data through a spatial phylogenetic model (see Materials and Methods in the article main text for details). Colors and legend details are identical to those in Figure 2 and 3 in the main text.

A

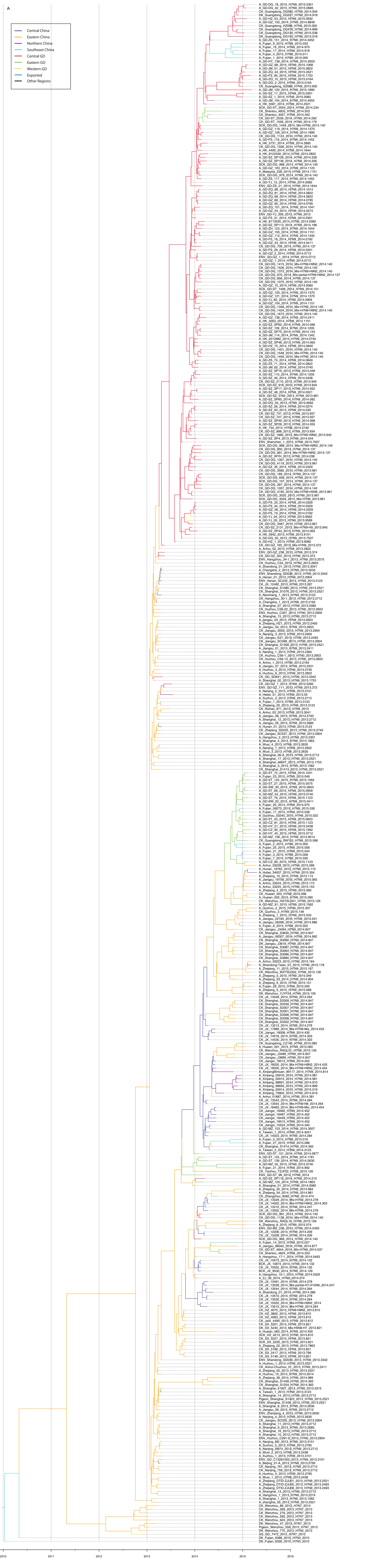

Central China
Eastern China
Western China
Southeast China
Central GD
Eastern GD
Western GD
Exported
Other Regions
2010
2011
2012
2013
2014
2015
2016

B

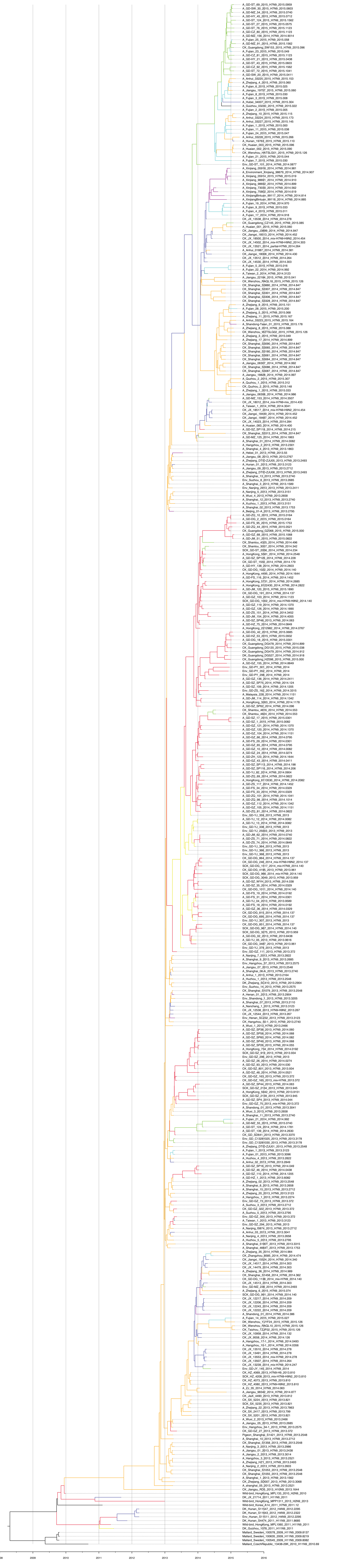

c

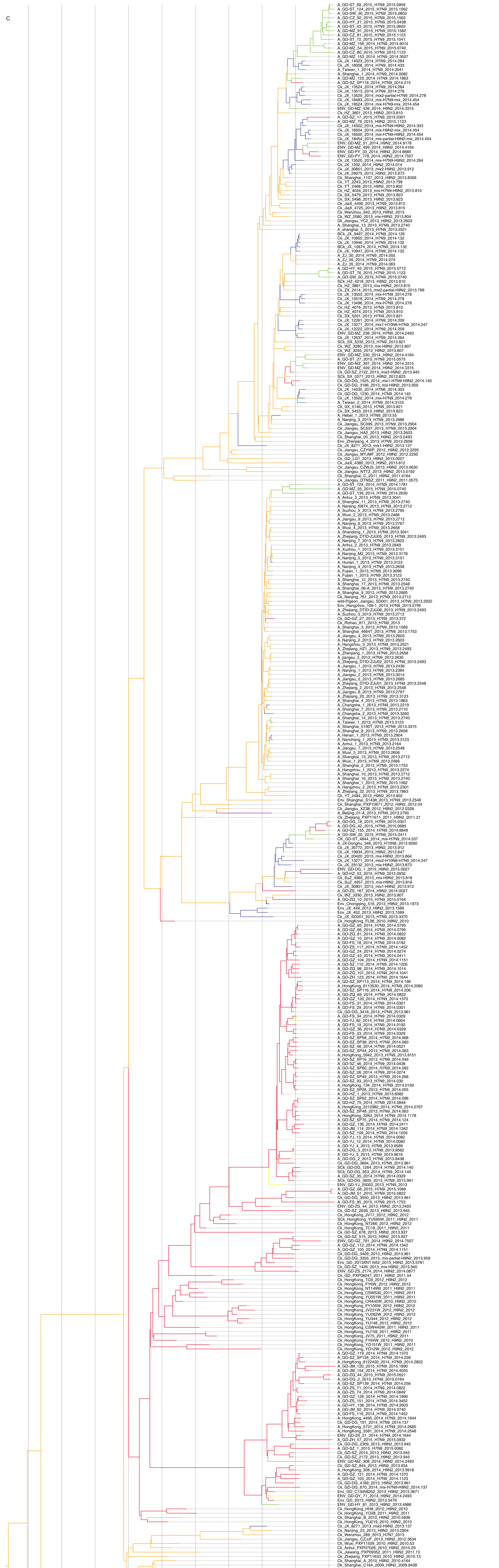

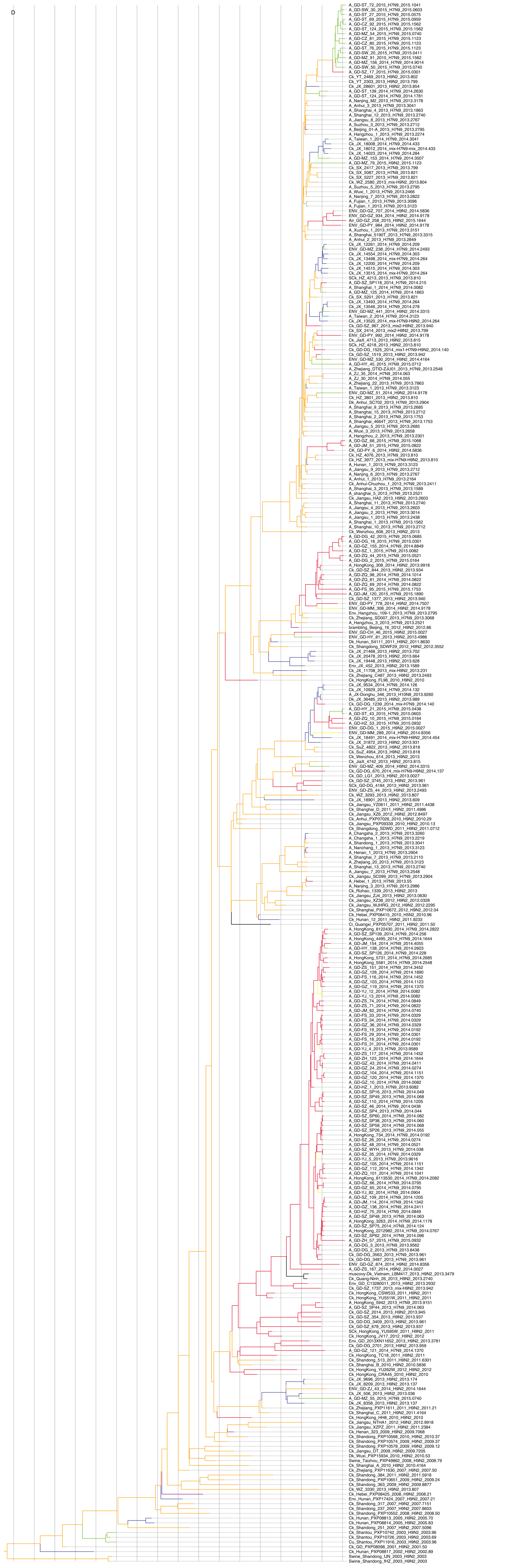
D
1999
2000
2001
2002
2003
2004
2005
2006
2007
2008
2009
2010
2011
2012
2013
2014
2015

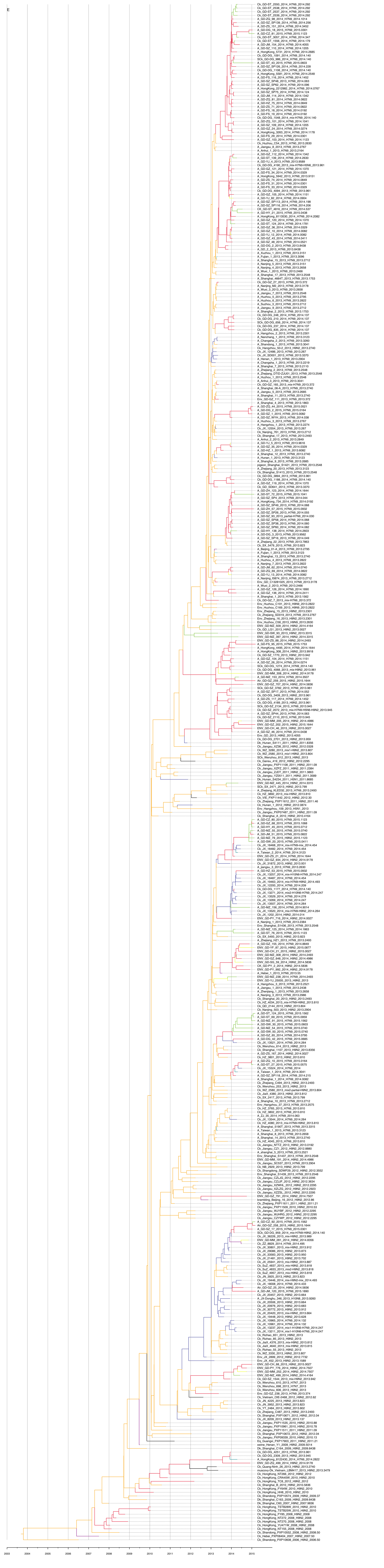
E
2003
2004
2005
2006
2007
2008
2009
2010
2011
2012
2013
2014
2015

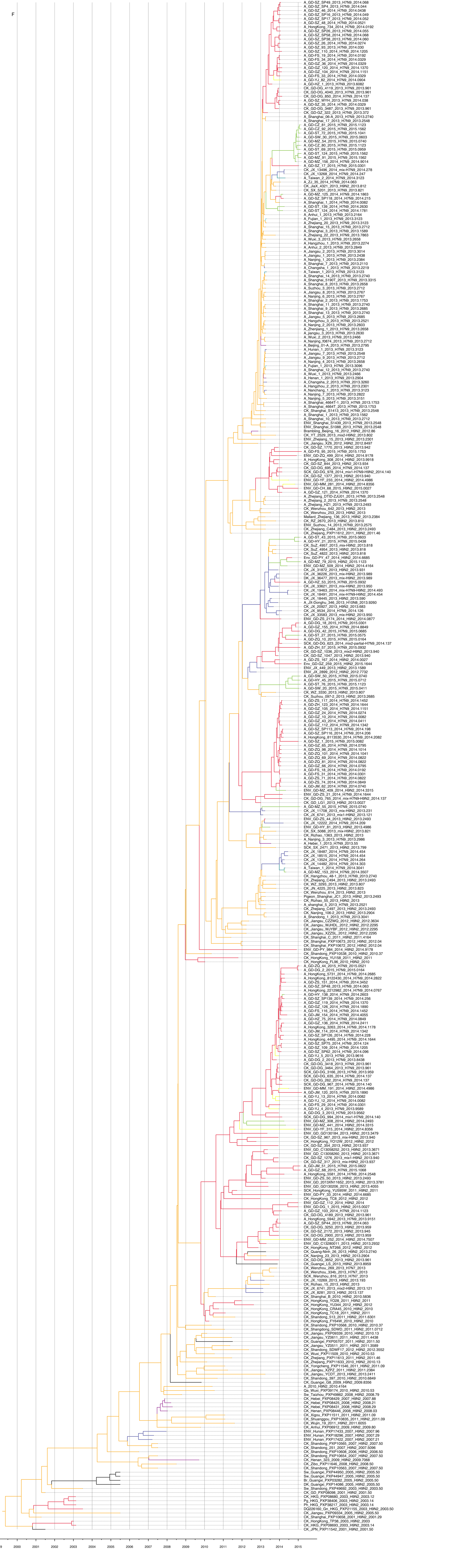
F
2000
2001
2002
2003
2004
2005
2006
2007
2008
2009
2010
2011
2012
2013
2014
2015

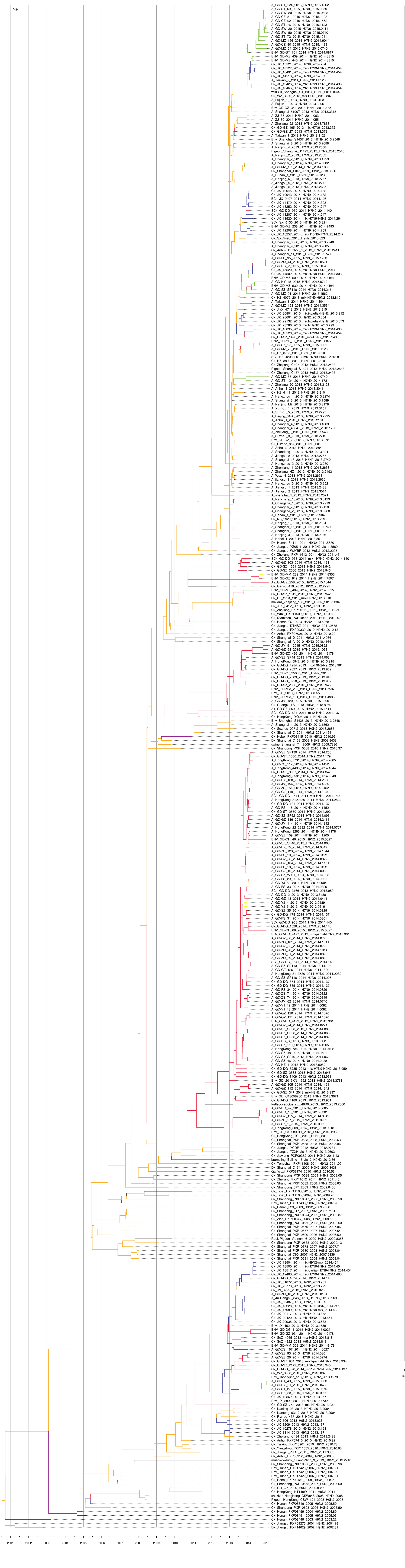
NP
2001
2002
2003
2004
2005
2006
2007
2008
2009
2010
2011
2012
2013
2014
2015

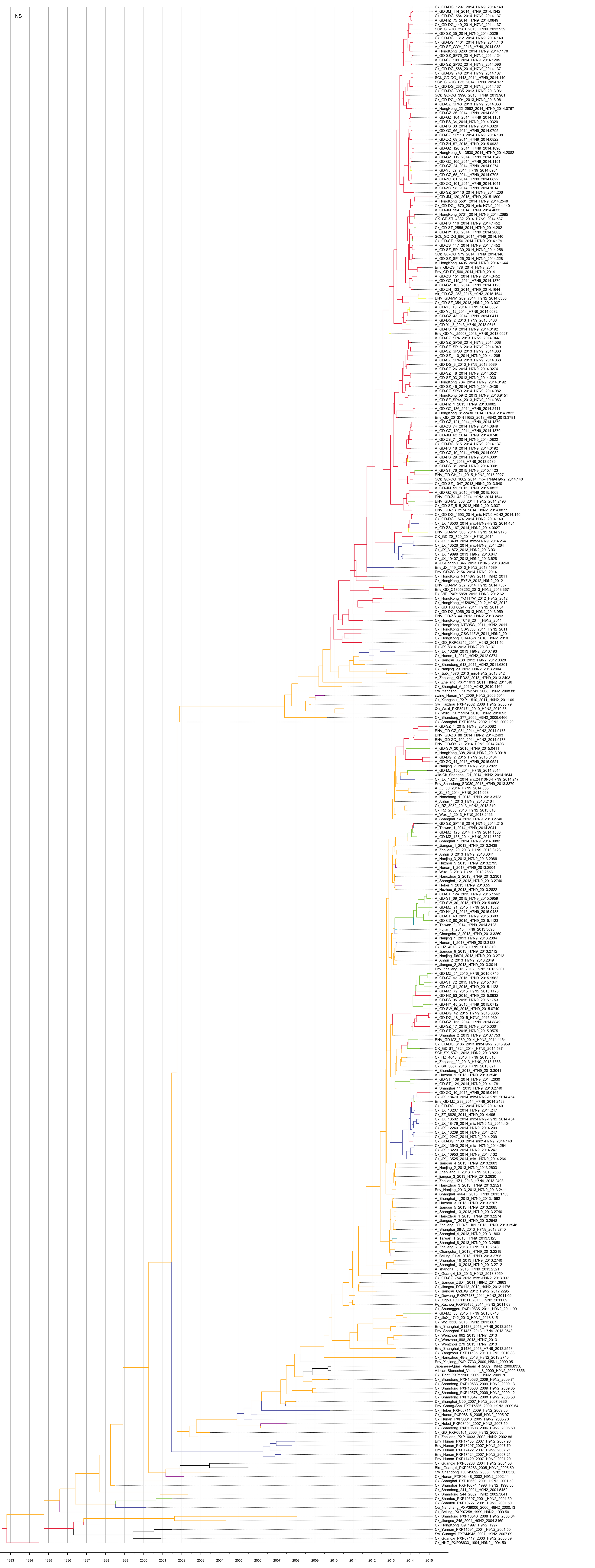
NS
1993
1994
1995
1996
1997
1998
1999
2000
2001
2002
2003
2004
2005
2006
2007
2008
2009
2010
2011
2012
2013
2014
2015
